# Anisotropic interaction and motion states of locusts in a hopper band

**DOI:** 10.1098/rspb.2023.2121

**Published:** 2024-01-17

**Authors:** Jasper Weinburd, Jacob Landsberg, Anna Kravtsova, Shanni Lam, Tarush Sharma, Stephen J. Simpson, Gregory A. Sword, Camille Buhl

**Affiliations:** ^1^ Mathematics Department, Hamline University, Saint Paul, MN 55104, USA; ^2^ Department of Mathematics, Harvey Mudd College, Claremont, CA 91711, USA; ^3^ Department of Physics and Astronomy, Haverford College, Haverford, PA 19041, USA; ^4^ School of Life and Environmental Sciences, University of Sydney, Sydney, New South Wales 2006, Australia; ^5^ Charles Perkins Centre, University of Sydney, Sydney, New South Wales 2006, Australia; ^6^ Department of Entomology, Texas A&M University, College Station, TX 77843, USA; ^7^ School of Agriculture, Food and Wine, University of Adelaide, Adelaide, Southern Australia 5005, Australia

**Keywords:** anisotropic interactions, collective motion, locust, swarm behaviour, hopper band, motion tracking

## Abstract

Swarming locusts present a quintessential example of animal collective motion. Juvenile locusts march and hop across the ground in coordinated groups called hopper bands. Composed of up to millions of insects, hopper bands exhibit aligned motion and various collective structures. These groups are well-documented in the field, but the individual insects themselves are typically studied in much smaller groups in laboratory experiments. We present, to our knowledge, the first trajectory data that detail the movement of individual locusts within a hopper band in a natural setting. Using automated video tracking, we derive our data from footage of four distinct hopper bands of the Australian plague locust, *Chortoicetes terminifera*. We reconstruct nearly 200 000 individual trajectories composed of over 3.3 million locust positions. We classify these data into three motion states: stationary, walking and hopping. Distributions of relative neighbour positions reveal anisotropies that depend on motion state. Stationary locusts have high-density areas distributed around them apparently at random. Walking locusts have a low-density area in front of them. Hopping locusts have low-density areas in front and behind them. Our results suggest novel insect interactions, namely that locusts change their motion to avoid colliding with neighbours in front of them.

## 1. Introduction

Locust hopper bands exhibit a striking example of collective motion in insects. Without a complex social structure, juvenile locusts self-organize into a range of patterns from columnar streams to planar fronts that appear to serve ecological functions for the group, such as migration and foraging [1,2]. Similar collective motion is observed in schools of fishes [3], flocks of birds [4,5] and even herds of ungulates [6]. These natural phenomena have produced a field of research centred on the idea that a group can attain collective goals without centralized instruction.

Most modern models of collective motion rely on a few rules for simple interactions between individuals, and so are fundamentally similar to their predecessors [7–9]. These interactions are often characterized as attraction, repulsion and alignment of the direction of motion. Empirical studies by biologists, ecologists and physicists have provided evidence supporting these models and informing these interaction rules for specific species, for instance, in starlings [10] and golden shiners (fish) [11]. Buhl *et al.* [12] have previously made observations for locust interactions, which we aim to build on here.

Locusts and their motion are the subject of studies ranging from empirical work [1,12–16] to theoretical modelling [17–19]; for a review, see the work of Ariel & Ayali [20]. Locust behaviour and motion is grounded in their biology. Locusts exhibit phase polyphenism, a phenomenon whereby an individual can exhibit two distinct phases of behaviour (and for some species also distinct morphologies). In the solitarious phase, locusts typically avoid each other and forage individually. Crowding by conspecifics triggers a transition to the gregarious phase in which individuals gather in social aggregations [21]. When composed of juveniles, these aggregations are called *hopper bands* because locust nymphs, whose wings have not fully developed, hop and walk across the ground. Hopper bands are often composed of hundreds of thousands of locusts all moving as a collective [1,16]. Researchers study how the persistence, motion and shape of a hopper band develop from the interactions between individuals through the lens of collective motion.

Relatively simple collective motion models have successfully reproduced collective patterns of hopper bands observed in the field. Dkhili *et al.* [17] demonstrated that both columns and fronts can be achieved by varying individual-level parameters in a model that incorporated only local interactions between locusts. A similar approach was taken by Bach [18], with a realistic number of individuals and parallel computing approach. An alternative approach by Bernoff *et al.* [19] incorporated individual locust interactions with food resources and showed that dense fronts are typical when sufficient food is present. Modellers often rely on hypothesized interactions at the individual level. The most common assumption is the simplest; that individual interactions are isotropic, that is, interactions depend only on the distance between individuals and not on their relative positions. One notable exception is the escape-and-pursuit model [22], which hypothesizes that proximate behavioural responses are the result of ultimate selection pressures related to cannibalism risk [14] (chasing those in front and fleeing from those behind). In this modelling framework, locusts are likely to have neighbours directly in front or behind them [23], but this has not been observed empirically.

A majority of the empirical work studying the motion of hoppers is conducted through either laboratory experiments focussed on individuals [13–15] or field observations of the group as a whole [1,12,16]. These field observations date back to Clark [16] and Ellis & Ashall [24] and are mainly qualitative in nature, for instance noting the shape of the hopper band at different times of day or in varying vegetation cover. Many laboratory studies are conducted by placing a relatively small number of locusts (less than a hundred) in an arena and observing their motion. While these empirical studies have advanced our understanding of the mechanics of locust motion and interaction, there is still a particular dearth of data on individual interactions during collective motion within a group of a naturally occurring size.

We present and analyse the first trajectory data of individual locusts moving within a hopper band. We study four hopper bands of the Australian plague locust (APL), *Chortoicetes terminifera*, from an outbreak in 2010 near Hillston, New South Wales, Australia. We recorded video of locusts moving across the ground using cameras mounted on tripods (see the electronic supplementary material, appendix A—*Sample video of marching locusts* for a sample). Using automatic tracking software (TrackMate [25]), we extracted 19 687 individual trajectories by linking 3 369 723 locust positions. Our analysis of these trajectories suggests that locusts adjust their motion to avoid neighbours ahead of them, providing evidence of a novel locust-locust interaction for collision avoidance. These results add to the understanding of individual interactions in marching locusts and provide valuable insight for modellers seeking to reveal the mechanisms behind the collective motion of the swarm.

## 2. Results

**Figure 1.**
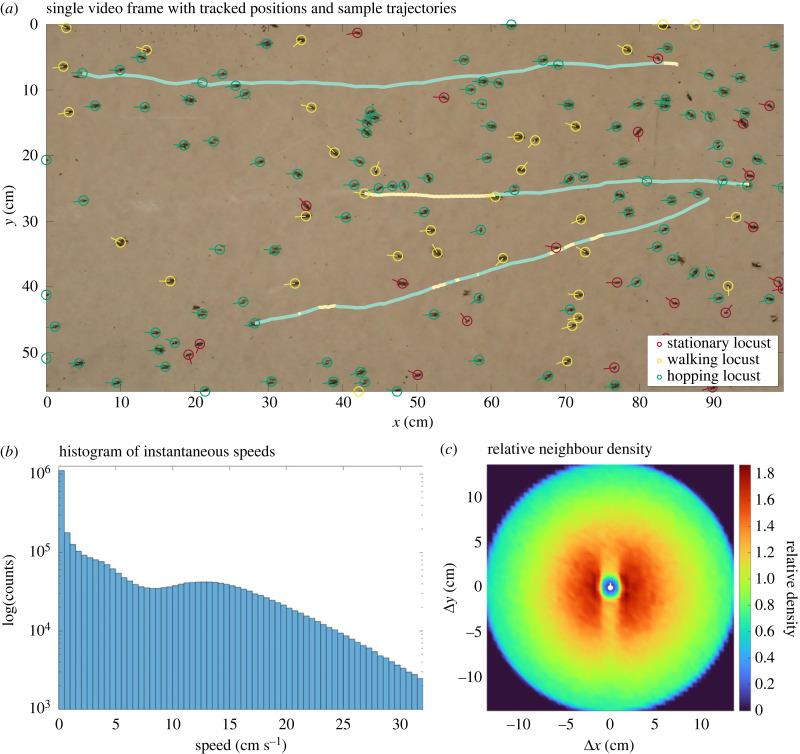
Single frame of video with processed data (*a*), histogram of speeds (*b*) and plot of relative neighbour density (*c*). The image (*a*) is taken from the video with processed data in the electronic supplementary material, appendix A and augmented showing the trajectories of three sample locusts with colour denoting the motion state. The distribution of speeds (*b*) is bimodal, with peaks near 0 and 13 cm s^−1^. From approximately 2−5 cm s^−1^, the counts decrease exponentially (linear decrease on the logarithmic scale). The relative neighbour density (right) is computed from 19 407 719 relative neighbour positions around a representative focal locust (white marker) positioned in the centre and oriented facing upwards, along the vertical axis. Density is highest (red) within a radius of 5 cm and lowest (blue) in a central disc with radius approximately 1 cm. The density is not rotationally symmetric; at distances of 1−7 cm, there is a noticeable decrease in density (orange) directly in front (above) and behind (below) the focal locust. At distances greater than 7 cm, the density appears to be rotationally symmetric.

Our dataset was extracted from recordings of four bands of APL, *C. terminifera*. The data consist of 3 369 723 locust positions linked into 19 687 trajectories from 24 300 frames (27 min) of video. Three sample trajectories are shown in figure 1*a* with a still image and processed data from the electronic supplementary material, appendix A. From all trajectories, we inferred a total of 3 332 137 heading directions and individual speeds; figure 1*b* shows a histogram of these speeds. We constructed a plot of the relative density around a focal individual from 19 407 719 nearby neighbour positions, shown in figure 1*c*. The anisotropy apparent in this plot contrasts with previous findings by Buhl *et al.* [23] and motivates deeper investigation. To this end, we classified the data into distinct motion states (stationary, walking and hopping) using statistical learning. We partitioned the relative neighbour data by motion state of the focal individual to reveal differences in anisotropy depending on motion. Of note, in the remainder of the paper, we often use ‘locust’ to refer to the APL specifically and acknowledge that some results may be species specific.

### (a) Collective marching

All four bands exhibited collective marching behaviour. The mean density across all recordings $141.6\;{\mathrm{locusts}\;m}^{-2}$ is well above the established 20 locusts m^−2^ threshold for marching [13]. Measurements of group alignment (polarization and entropy index) agree with previous measurements of marching locusts in the field [12]. Polarization is the length of the average of the direction vector and our entropy index is an adaptation of Boltzmann entropy, see §4d and the electronic supplementary material, appendix F for details. We compute a mean polarization of 0.82 and mean entropy index of 0.76 across all bands.

**Figure 2.**
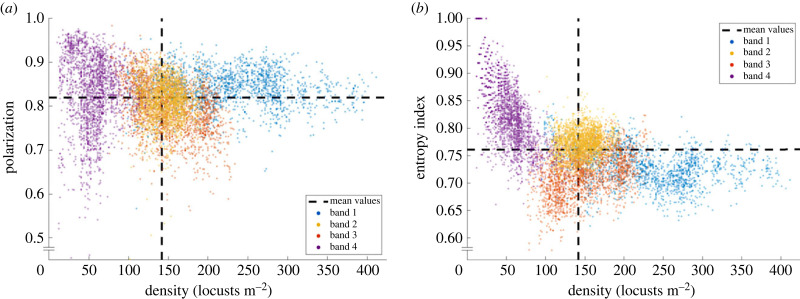
Measures of collective alignment plotted against density for all bands (distinguished by colour). Polarization (*a*) is highly variable for densities less than 200 locusts m^−2^. Entropy (*b*) shows a marked decrease with increasing density, consistent with [12]. Data plotted are a regular subsample of frames (every fifth frame), with mean values of the full dataset (dashed lines). Essentially all measurements lie within ranges associated with collective marching for hopper bands.

We present values for each band in the electronic supplementary material, appendix B table S2 and direct measurements for a regular subsample of frames (every fifth frame) as scatter plots in figure 2. The density of each band is relatively well-clustered, as expected. There is a distinct decrease in entropy with increasing density and a high variance in polarization for densities below 200 locusts m^−2^. See [12] for an in-depth analysis of similar trends.

### (b) Individual speed and motion state

Of the 3 369 723 locust positions, we computed 3 332 137 individual speeds, presented in figure 1*b*. (The discrepancy in the number of positions and speeds is owing to the start and end of trajectories.) Each speed was classified into one of three motion states by a support vector machine (SVM). Our classification uses four summary statistics computed in a moving time window; further details are provided in §4e. These motion states divide the data into 26.0 % stationary, 24.7 % walking and 49.2 % hopping. While there is some variation between bands in the composition by motion state, this variation does not appear to be correlated with density or group alignment. We computed mean speeds for each motion state and found (0.2 ± 0.7) cm s^−1^ (stationary), (2.7 ± 2.2) cm s^−1^ (walking) and (11.8 ± 9.3) cm s^−1^ (hopping). Each mean speed has plus/minus one standard deviation. Motion state and speed data are presented for each band in the electronic supplementary material, appendix B table S3. See the electronic supplementary material, appendix A—*Sample video with tracking data* for a visualization showing results of the motion state classification.

**Figure 3.**
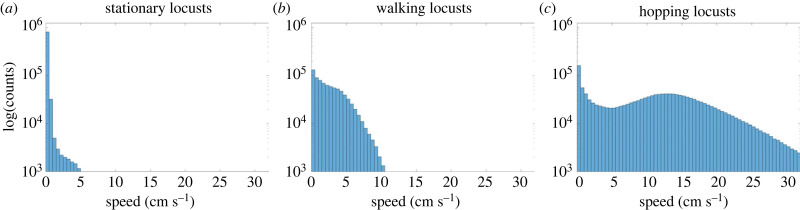
Histograms of speeds for stationary (*a*), walking (*b*), and hopping (*c*) locusts. All appear with the same log-scaled vertical axis. Matching intuition, the speeds of stationary locusts are tightly grouped near 0 cm s^−1^ (speeds above 1 cm s^−1^ make up less than 2.5% of all stationary data). Walking locusts have speeds between 0 and 5 cm s^−1^ with a steeper decline in numbers after 5 cm s^−1^. Hopping locusts have a bimodal distribution with one peak near 0 and the other near 13 cm s^−1^, as we expect from the pattern of pausing between hops observed in our recordings.

Figure 3 shows histogram plots for the speed distributions divided by motion state. Note the logarithmic scale on the vertical axis. Speeds of stationary locusts essentially all fall into the first two bins 0−1 cm s^−1^. Higher speeds make up less than 2.5% of all data classified as stationary. The number of walking speeds have no such dramatic peak near 0, instead decreasing slowly until 5 cm s^−1^, then decreasing super-exponentially (concave down on the logarithmic plot). Speeds of hopping locusts are by far the most widely distributed with two peaks at 0 and 13 cm s^−1^, matching our observation that hopping locusts often pause briefly between two jumps. A minimum around 5 cm s^−1^ separates these two maxima and the hopping speeds decrease super-exponentially after the second. Comparing these three plots to figure 1*b*, we observe that our motion state assignment has cleanly divided the data into three distributions with unique features that were each visible in the full distribution.

### (c) Anisotropy in relative neighbour density

Figure 1*c* demonstrates that the relative neighbour density is not isotropic, i.e. not rotationally symmetric, particularly at distances of 1−7 cm from the focal individual. At distances greater than 7 cm, the density appears isotropic. We focus next on a smaller square around the focal locust so as to exclude the isotropic region at larger radii.

**Figure 4.**
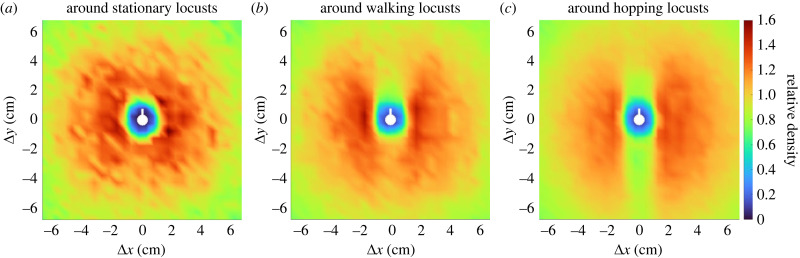
Relative neighbour density around locusts that are stationary (*a*), walking (*b*), and hopping (*c*). A representative focal locust (white marker) is positioned in the centre of each plot and oriented facing upwards along the vertical axis. Areas of highest density (red) are distributed apparently at random around a stationary focal locust but highly anisotropic around walking and hopping focal locusts. A notable sector-shaped area of lower density (green-orange) lies immediately in front (above) the walking focal locust. A longer and narrower strip-shaped area of low density (green) lies in front and behind the hopping focal locust.

In figure 4, we plot the relative neighbour densities around focal locusts that are stationary, walking and hopping. In each plot, the focal locust is positioned in the centre (Δ*x* = Δ*y* = 0) and faces upwards (along the Δ*y*-axis). The highest relative neighbour densities are indicated by red, intermediate densities are shown in green and the lowest neighbour densities appear in blue. For all three motion states, there is a roughly circular area of low density around the focal individual with a radius of approximately 1 cm. Past a radius of 7 cm, the plots are roughly isotropic (rotationally symmetric) and similar between motion states.

The visual differences in relative density plots between motion states in figure 4 are striking. For stationary focal locusts, the relative neighbour density is mostly isotropic, with localized spots of high density (red) distributed apparently at random angles around the focal locust. For walking locusts, there is a distinct area of lower density ahead of the focal individual. This void has the approximate shape of a 45°-sector. The highest relative neighbour densities are directly to the left and right of the focal individual at a distance just less than 2 cm. For hopping locusts, there is a strip-shaped area of low density ahead and behind the focal individual. This void appears to divide an otherwise circular area of high density that decreases with distance from the centre. The high-density area to the right of the focal individual is larger than on the left, which we attribute to a density gradient in recordings of bands 1 and 3. The observed low-density sector in front of walking locusts and the strip both in front and behind hopping locusts are novel anisotropies for neighbour densities in the APL and other locust species.

We quantified these anisotropies by examining the angular distribution of neighbours within 7 cm. First, the Hodges-Ajne test for uniformity confirms that, for a focal locust in any motion state, the distribution of neighbour angles is not drawn from the uniform distribution of angles; we compute *p*-values less than e^−10^ (stationary), less than e^−128^ (walking) and less than machine error (hopping). We characterize the degree of this non-uniformity by computing trigonometric moments $M_{p}=\langle M_{\;p}^{c},M_{\;p}^{s}\rangle$, which we record in the electronic supplementary material, appendix B table S4. Overall anisotropy |**M**_*p*_| is significantly larger for the distributions around walking and hopping locusts (|**M**_1_| = 0.0173, 0.0203 and |**M**_2_| = 0.0248, 0.0358) than around stationary locusts (|**M**_1_| = 0.0040 and |**M**_2_| = 0.0146). This quantitatively confirms what figure 4 shows visually—that the distributions around moving locusts are less isotropic than around stationary locusts.

In figure 4, we noted two forms of anisotropy around moving locusts. First, the sector of lower density in front of walking locusts is unimodal and therefore measured by **M**_1_. We compute the *front-back asymmetry* as $-M_{1}^{s}=M_{1}\cdot \langle 0,-1\rangle$ to measure its size along the apparent axis of asymmetry (vertical). The negative captures the lower density in front and higher density behind. We point out the extreme disparity in $-M_{1}^{s}$ between the distributions around walking (0.0173) and stationary (0.0023) focal locusts. Second, both walking and hopping locusts have areas of high density on either side. This is measured by **M**_2_ and we compute the *fourfold anisotropy* $M_{2}^{c}=M_{2}\cdot \langle 1,0\rangle$ to measure its size along the apparent axis of high density (horizontal). We highlight the extreme disparity in $M_{2}^{c}$ between the distributions around hopping (0.0357) and stationary (0.0142) focal locusts. These values are reported in bold in the electronic supplementary material, appendix B table S4, along with other detailed anisotropy quantities.

**Figure 5.**
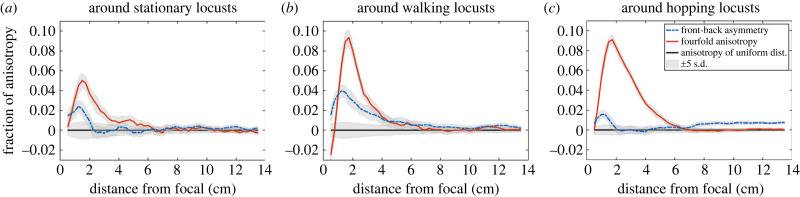
Anisotropy around stationary (*a*), walking (*b*), and hopping (*c*) locusts as functions of distance from the representative focal individual. Each plot shows front-back asymmetry $-M_{1}^{s}$ (dashed blue), fourfold anisotropy $M_{2}^{c}$ (solid orange) and anisotropy computed analytically for a uniform distribution $-M_{1}^{s}=M_{2}^{c}=0$ (black). Grey shading represents ±5 s.d. Both measures of anisotropy around stationary locusts are approximately half of the same measure around moving locusts and decrease quickly towards uniformity. Front-back asymmetry (dashed blue) peaks before decreasing slowly around walking locusts, while it is generally smaller around hopping locusts. Fourfold anisotropy (solid orange) around both walking locusts and hopping locusts peaks at a radius of 2 cm, then decreases quickly around walking locusts and slowly around hopping locusts. At distances above 8 cm, there is small but significant front-back asymmetry around hopping locusts.

Finally, in figure 5, we examined how front-back asymmetry $-M_{1}^{s}$ (dashed blue) and fourfold anisotropy $M_{2}^{c}$ (solid orange) depend on distance from the focal individual. We computed $-M_{1}^{s}$ and $M_{2}^{c}$ for subsets of neighbours from overlapping annuli of width Δ*r* = 1 cm at intervals of *r* = 0.25 cm. In each annulus, we normalized the anisotropy by scaling with the ratio of the density in that annulus to the density in the complete disc with radius 14 cm. For a measure of distance from uniformity, we plotted the same quantities computed analytically for a uniform distribution. The uniform distribution of angles on the circle has $-M_{1}^{s}=M_{2}^{c}=0$ (black) and standard deviation $1/\sqrt{2N}$, where *N* is the number of neighbour positions in the current annulus. Grey shading represents ±5 s.d.

For a given distance, both measures of anisotropy around stationary locusts (*a*) are small—approximately half—compared to the same around moving locusts (*b*,*c*). Front-back asymmetry $-M_{1}^{s}$ (dashed blue), is more than twice as large around walking locusts (*b*) than around either other motion state. Smaller front-back asymmetry around hopping locusts at short distances may be attributed to their high movement speeds. Specifically, any neighbours or open spaces ahead of them will be behind them a fraction of a second later; our time-aggregated plots manifest this as front-back symmetry around hopping locusts. Fourfold anisotropy $M_{2}^{c}$ (solid orange) peaks around both walking locusts and hopping locusts (*c*) at a radius of approximately 2 cm, but decreases quickly around walking locusts and more slowly around hopping locusts. Both anisotropy quantities decrease to near 0 at a distance between 6 and 8 cm for all motion states. Around hopping locusts, we note a small increase in front-back asymmetry for distances greater than 8 cm. Values for distances smaller than 0.5 cm are computed from relatively few neighbours, resulting in the initial negative value of $M_{2}^{c}$ around walking locusts. These computations quantify our visual observations of figure 4. Moreover, they reveal lengthscales where the anisotropies are strongest.

## 3. Discussion

Insight into animal collective behaviour develops from the feedback between theoretical studies of universal models and empirical studies of the animals themselves. The modelling approach seeks to reproduce a range of incredible structures, patterns and group strategies based on simple and often local interactions between individuals. Meanwhile, the empirical work aims to uncover the specifics of individual-level behaviours. One challenge of conducting empirical studies has been to capture individual behaviour in the midst of large and often dense groups of animals. This has been a particular difficulty for locusts owing to the rarity of their swarming, small size and disorder compared to larger animals. Identifying coherent signal amidst this inherent noise necessitates a quantity of data orders of magnitude larger than similar studies of other species.

The fine timescale of our trajectory data (comprised of almost 20 000 individual locust trajectories, resolved at 25 frames s^−1^) allowed the first analysis of individual locust motion in the field. We classified motion into three distinct modes: stationary, walking and hopping. Intermittent motion was previously quantified in laboratory studies of different locust species (the desert locust) [15], and has typically been considered a binary between ‘stop’ and ‘go’. Our evidence suggests a significant difference between the two moving states (walking and hopping) for the APL. In theoretical models, intermittent motion provides a collective mechanism for density regulation [13,17,19]. By including hopping in future models, our findings may help to reproduce the dense fronts that occur in hopper bands of the APL. Our data have revealed novel anisotropies in the positions of neighbours around focal locusts in a natural setting. In particular, we find a lower likelihood of frontal neighbours around moving (walking and hopping) focal locusts. This represents a significant advance over the only other study that examined interactions of APL within a hopper band [23]. Relying on manual data collection, that study computed a rotationally symmetric distribution from less than 20 000 neighbour positions in a disc of radius greater than 28 cm. Automated particle tracking enabled us to collect nearly 20 000 000 neighbour positions in a disc of radius 14 cm. After accounting for the difference in area, this represents an increase in number of data points by four orders of magnitude. In turn, this revealed the previously invisible anisotropies now clearly apparent in figure 4.

These anisotropies have not been produced by models of locust interaction and hopper band collective motion. Previous models of locust interaction are either isotropic [17,18] or based on the idea of escape-and-pursuit [22]. As shown explicitly by Buhl *et al.* [23], the escape-and-pursuit paradigm generates an anisotropy in neighbour density where localized high-density areas appear in front and behind the focal locust. This contrasts with the anisotropy found in our analysis. Models with isotropic interaction produce isotropic neighbour densities, also contrasting with our findings. New mechanisms of locust interaction are probably necessary to emulate our findings. One possible explanation for our anisotropy results could be external factors such as prevailing winds or orientation of the sun. However, direct field observations have noted no correlation between a band’s direction and either of these [16,24]. A second possible explanation could be that locusts themselves do not have an isotropic shape. Typically, locust bodies are much longer (head to end of abdomen) than they are wide. In a recent study of the desert locust, Gorbonos *et al.* [26] report that relative density distributions become isotropic after applying a correction to nearby neighbour data. By contrast, our anisotropy results for the APL persist under similar corrections; see the electronic supplementary material, appendix G for details. The simplest interpretation for these differing results is that the individual interactions differ between these two species of locust.

### (a) Anisotropies in other animals

Most quantitative studies of animal groups reveal structure in the position of nearby neighbours. In contrast to our findings for locusts, higher densities are observed ahead and behind the focal individual in the Serengeti wildebeest [6], surface-swimming surf scoters [5] and some species of fishes [11]. This type of anisotropy is typically associated with following behaviour. Various species of fish display a range of likely relative neighbour positions, including at a diagonal or with no preference [27] and laterally [3,28]. Starlings keep their nearest neighbours on their left and right, but this preference fades when considering all neighbours in a given radius [10].

We found an area of lower density ahead of moving focal locusts, see figure 4*b*,*c*, which is a unique feature when compared with other species. Quantifying this in the front-back asymmetry, figure 5 (dashed blue curve), we established its presence at distances less than 7 cm around walking locusts and at distances less than 2 cm and greater than 7 cm for hopping locusts. Simultaneously, we found that distributions of neighbours around moving locusts exhibit a fourfold anisotropy similar to that of starlings, with lateral areas of high density; see figures 4*c* and 5 (solid orange curve). Various explanations have been given for the higher occurrence of lateral neighbours for airborne birds and some fishes. Ballerini *et al.* [4] suggest that this fourfold anisotropy could be owing to a hydro/aerodynamic advantage, anisotropic vision, or a mechanism to avoid collisions in a high-speed group. Since these juvenile locusts do not fly, they would derive no significant aerodynamic benefit. Since locusts have essentially 360° vision [29], limited sight lines are not a likely explanation. Given the additional presence of front-back asymmetry around moving locusts, we suggest that the mechanism causing the anisotropies revealed here is one of collision avoidance.

Collision avoidance has been observed in other species exhibiting collective behaviour. Above we noted it as a potential mechanism explaining the relative position of lateral neighbours in starlings [4]. In surf scoters [5], where high-density areas were found directly in front and behind focal individuals, examining the deviation from the mean velocity in the presence of frontal neighbours revealed that these ducks tend to follow frontal neighbours with a preferred following distance, slowing down to avoid collisions. Similarly, Katz *et al.* [11] found that golden shiners modulate their speed to avoid collisions while following neighbouring fish. These following behaviours are among the best-documented examples of collision avoidance in collective behaviour that we are aware of, but are qualitatively different from the case of locusts. Locusts appear to have far less control over their speed, probably owing to the incredibly high strength to mass ratio characteristic of insects. We hypothesize that they instead tend to change their direction or type of motion to avoid collisions.

### (b) Collision avoidance in locusts

Collision avoidance behaviours are well-documented through laboratory experiments in adult locusts; see a review by Fotowat & Gabbiani [30]. Both neural and behavioural responses occur from visual looming stimuli [31,32] and have been particularly associated with collision trajectories [33,34]. Stimuli in these studies had an area of at least 5 cm × 5 cm and responses were associated with a particular size of retinal image on the eye of the locust [31]. Response behaviours have included avoidant gliding for tethered flying locusts [35] and jumping away from an incoming stimulus for locusts on the ground [36]. This body of work establishes that adult locusts have the physiology necessary to sense nearby obstacles and react to avoid collisions. Our empirical findings suggest that juvenile locusts may exhibit similar visual sensing and motion adjustment; in our case, in the midst of a naturally occurring swarm, albeit while marching en mass on the ground rather than in flight. It is unclear whether this behaviour may be owing to the same neural circuits well-studied in adult locusts, opening up new behavioural and neurological questions.

### (c) Recommendations for locust models of collective motion

Models of hopper band movement continue to advance towards the capability to predict the direction and distance that a given band will travel. The aim of such predictive models is to inform efficient control strategies for agricultural industry and government management agencies, possibly in real time. Predicting the likely trajectory and collective momentum of a threatening band can aid in conducting efficient surveys or implementing direct control strategies, such as pesticide barrier spraying [37]. Our results reveal state-dependent elements of locust motion and interaction during marching that are yet untested by current models and provide promising explanations for collective structure.

To start, we suggest a three-state model for capturing locust motion. The high level of accuracy (estimated at 85.0%) of our method for classifying motion states supports this framework. Moreover, each motion state comprised a significant fraction (nearly 25%) of the data derived from locusts naturally marching in the field. This makes it difficult to justify omitting any one motion state from a realistic model. Additional evidence comes from the clean division of our distribution of individual speeds shown in figure 3. A possible implementation could use a discrete time Markov process to dictate switches between stationary, walking and hopping states. Going further, our distributions of speeds for each state could add biologically realistic individual variation to such a model. Secondly, we suggest modelling locust interactions with mechanisms for collision avoidance. By contrast, existing models of locust interaction either treat equidistant neighbours the same (i.e. are isotropic) or implement the escape-and-pursuit paradigm [14,22] (where motion is driven by chasing behaviour). As noted above, we find that moving locusts (both walking and hopping) have a high density of neighbours on the left and right. A first possible mechanism for this anisotropy might be to implement a preference for lateral neighbours. However, this alone would probably not explain the additional front-back anisotropy we observe around moving locusts.

The area of lower neighbour density immediately in front of walking locusts (figure 4*b*) suggests that when walking locusts see another individual in front of them, they react to avoid a direct collision. This behaviour could appear in a model by using some anisotropic kernel function when updating heading direction according to nearby neighbours and a lengthscale could be drawn from figure 5*b*.

The anisotropy around hopping locusts (figure 4*c*) suggests a slightly different mechanism. Indeed, juvenile hoppers cannot change their motion mid-jump. Noting that the low-density area ahead of hopping locusts is narrower and more elongated than ahead of walking locusts, we suggest that one possibility is that locusts visually inspect a long and narrow area ahead of them before hopping and are more likely to hop when the path ahead is clear. Supporting this suggestion, there is a slight increase in front-back anisotropy for distances greater than 7 cm ahead of hopping locusts, see figure 5*c*. This lengthscale also provides modellers with a valuable parameter when implementing such a mechanism, which could be encoded into a front-neighbour-dependent probability to switch out of the hopping state.

Recent modelling studies by Taylor *et al.* [38] and Krongauz & Lazebnik [39] have specifically noted the impact collision avoidance interactions can have on collective behaviour. We hope that future models of collective motion in locusts will incorporate some of these specifics in order to determine their effect on the collective structure and function of the hopper band. Such predictions at the band level can then be tested in future field studies.

### (d) Further refinements

There are two limitations of our study that bear acknowledgement and provide opportunity for further investigation. The first is that our particle tracking implementation only identifies locust position. Consequently, we must infer heading direction from velocity. Since locusts move forward, almost never backwards or sideways, we are confident in the assumption that body orientation is equivalent to heading direction, i.e. direction of motion. The remaining issue is that stationary locusts do not have a well-defined heading direction. Via smoothing and interpolation we assigned a heading direction where we could confidently do so, but applying particle tracking software that collects body orientation, such as the newest release of TrackMate [40], may be preferable.

Secondly, we employed a supervised classification method for determining motion state. While we achieved a high degree of accuracy, a classification that makes use of unsupervised learning might uncover additional motion states that are not immediately apparent to the human eye while watching the recorded footage. For instance, only after watching footage in detail did we begin to note non-moving locusts rotating their body’s orientation without advancing in any direction. This often occurred after a locust made an especially large jump. Particularly for small hoppers, these large jumps ended with a crash landing so that the insect’s orientation was no longer aligned with the prevailing direction of the band’s motion. The locust would rotate to align itself with passing neighbours before hopping again. Especially in conjunction with tracking software that records body orientation, identifying this behaviour as distinct from other hopping might provide even stronger evidence of collision avoidance or novel patterns of interaction.

### (e) Towards predictive modelling

In addition to informing theoretical models of collective behaviour, our empirical investigation of individual mechanisms contributes to the development of a well-parametrized predictive locust movement model. With the addition of our findings, we are ever closer to predicting collective band behaviour from evidence-based mechanisms for individual motion and interaction.

## 4. Methods

### (a) Recording hopper bands

We recorded video footage of eight distinct hopper bands of APL, *C. terminifera*, during 3–10 of November 2010 near Hillston, New South Wales, Australia. The Australian Plague Locust Commission directed us to the area and put us in contact with the local control agency in Hillston who then took us to potential study sites. Site locations were 33.54733 S, 145.06678 E (for bands 1–3) and 33.20745 S, 145.09763 E (bands 4–8). The hopper bands were composed of late-instar juveniles (3rd to 5th). Of the eight bands recorded, we analysed four for this study.

Our recording procedure was similar to that described by Buhl *et al.* [12]. We mounted a camera on a tripod so that it pointed vertically downwards, with a view angle approximately perpendicular to the ground. We extended the tripod’s central column so that its legs did not obstruct the field of view. This resulted in a recorded area of the ground approximately 0.6 m^2^ using a Panasonic camcorder which recorded in 1080i.

For most recordings, we placed the tripod in the centre of a marching band. Placing the tripod often caused a temporary disturbance, which we allowed to dissipate before beginning recording once the natural flow of marching had resumed. In one case, for band 1, when the location of the hopper band was known and accessible, we set up the tripod ahead of the band allowing for a full recording of the centre of the band from front to back. For consistency, in this case, we analysed footage from after the front had passed the camera.

For each band, we chose the recording area to be flat and devoid of vegetation. We used areas located away from major obstacles that could prevent or impede locust marching such as trees, creeks and patches of dense vegetation. We placed a sheet of plywood in the camera’s view frame to provide a uniform background. We recorded the scale by placing a ruler in the field of view at the beginning of the video, or else by the known dimensions of the plywood (120 × 60 cm).

For the purposes of this study, we selected four recordings where we observe sustained marching. Each video has a resolution of 1920 × 1080 pixels and consists of 25 interlaced frames s^−1^. For this study, a total of 27 min of footage was analysed, representing 24 300 frames. For a sample of our video footage, see the electronic supplementary material, appendix A.

### (b) Extracting numerical trajectories (via motion tracking with TrackMate)

We analysed the footage using particle-tracking software TrackMate [25], a plugin for ImageJ. This software includes a suite of established particle detection and trajectory-linking algorithms, along with a user-friendly GUI and interoperability with Matlab for scripted batch tracking. For more on particle-tracking software and to see how an early version of TrackMate performed, see [41]. For full details on our video processing, tracking algorithms and parameters, and how we evaluated TrackMate’s accuracy, see the electronic supplementary material, appendix C.

### (c) Inferring motion

From the trajectory data for each locust, we inferred instantaneous velocity and decomposed it into speed and heading direction. To accurately compute these quantities, we first cleaned the trajectory data including a spatial transformation to correct for the camera angle and smoothing the position data. For more details on data cleaning, see the electronic supplementary material, appendix D.

After smoothing the position data, we computed velocities using a central difference method. Speed was calculated as the magnitude of the velocity. Taking the angle of the velocity in a standard coordinate system (*x*-axis to the right, *y*-axis up) we calculated heading direction. Note that heading direction is not well-defined for an unmoving locust. In fact, small fluctuations in position (inherited from automatic tracking) can produce large fluctuations in the heading direction. To account for this, we used linear interpolation to recompute the heading direction for stationary locusts after we classified their motion states.

### (d) Measuring collective marching

Since we wish to analyse the behaviour of individuals during marching, we compute three quantities that are associated with this collective behaviour. In each frame of video, we compute the density *D* by counting the number of locusts detected and dividing by the physical area in the frame. Typically, a density greater than 20 locusts m^−2^ has been associated with marching [13,42]. We also compute the polarization *P* as the length of the average of the direction vectors $\left\langle \cos\phi_{i},\sin\phi_{i}\right\rangle$, where *ϕ*_*i*_ is the heading direction of the *i*th locust. Polarization is a commonly-used order parameter ranging from 0 (completely disordered) to 1 (completely aligned). Marching locusts have demonstrated polarization values between 0.6 and 0.9 [12]. To supplement polarization, we also compute an index that captures an adaptation of Boltzmann entropy. This entropy index *E* varies from 0 (completely aligned) to 1 (completely disordered) and was originally described by Baldassarre [43]. For marching locusts, the same entropy index has been reported between 0.75 and 0.9 [12] and we follow the same implementation therein. For details on formulae or computations of density, polarization, or entropy index refer to the electronic supplementary material, appendix F.

### (e) Classifying motion state

Watching the raw footage, we observed locusts moving in one of three distinct motion states:

(i) stationary: locusts do not advance in any direction, may rotate their body’s orientation;
(ii) walking: locusts advance relatively slowly without leaving the ground, with short pauses separated by approximately 1 s of motion; and
(iii) hopping: locusts advance quickly through erratic jumps, sometimes with pauses of up to approximately 1 s between jumps.

Distinct motion states have been previously recognized in locusts, including all three of these [1,15,24]. We manually classified all locusts in the two manually-tracked 10 s clips, creating our training and test data for automatic classification.

We implemented a SVM to automatically classify each locust in each frame into one of the three motion states. SVMs are popular classification tools from machine learning—see the book by James *et al.* [44, ch. 9] for an accessible introduction. SVMs compute an optimal boundary between each class of data points in a training dataset, then use those boundaries to classify the remaining data. The boundaries depend only on the data points closest to them, called the *support vectors*, so the method is not sensitive to variations in well-classified data points. For our application, we used a nonlinear boundary via a Gaussian kernel function. Computing the boundary amounts to an optimization problem where misclassified data points are assigned a penalty value based on their distance from the boundary in a higher-dimensional space. We implemented our SVM using Matlab’s fitcecoc() function.

After initial exploration of the data, we chose four trajectory features to classify locust motion states in each frame. For each locust in each frame of video, we centred a time window on the current frame and computed the following summary statistics for classification:

(i) the instantaneous speed (after the smoothing described in) distinguishes locusts that are stationary at the current time;
(ii) the magnitude of average velocity distinguishes a stationary locust from one that has paused between jumps;
(iii) the standard deviation of speed distinguishes between hopping (fast and intermittent speed) and walking (slow and almost constant speed); and
(iv) the minimum of the forward and backward maximum speeds helps to discern between stationary and hopping locusts near the beginning or end of their trajectories in these states.

We computed the latter three using a moving time window of 15 frames, equivalent to 0.6 s. The time window was chosen by tuning the classification accuracy of our SVM on the training data. To avoid overfitting, we optimized various internal hyperparameters of our SVM using cross-validation on the training data. We trained the SVM using the same dataset and found that it correctly classified 86.8% of the training data. We then evaluated the accuracy of our SVM on the second ground-truth dataset and found correct classification for 85.0% of the test data. Given the magnitude of the whole dataset, this represents a level of accuracy that should easily distinguish trends from noise. See the electronic supplementary material, appendix A for a sample video that illustrates the accuracy of our motion classification.

### (f) Quantifying anisotropy in neighbour density

Using the position and heading direction data, we aggregated relative positions of neighbours into density distributions using a methodology similar to Buhl *et al.* [23]. For each focal locust in a frame, we computed the relative position of each neighbour as its position in a coordinate frame with the focal individual at the origin, the *y*-axis pointing from tail to head, and the *x*-axis protruding to its right. We avoid biases introduced by the edges of the frame by taking two precautions. First, we only considered neighbours within 14 cm of the focal locust. This distance includes the only estimate we know of for the interaction range between locusts, which is 13.5 cm [23]. Second, we used the Hanisch correction [45,46], which ignores any neighbour at a distance further from the focal individual than the nearest edge. We discretized this distribution of relative neighbour positions into square bins with side length Δ*x* = Δ*y* = 0.5 cm. We normalized the counts in each bin by the average density over all focal locusts divided by the average density in the whole area. The effect of this normalization factor is that as the distribution approaches homogeneity, the value in each bin approaches 1. We call these distributions *relative neighbour densities* and plot them as two-dimensional maps where colour indicates density, see figures 1*c* and 4. This relative density can equivalently be thought of as the likelihood of finding a neighbour in a given position relative to a focal individual.

We also extracted the angle of each relative neighbour position and examined these as a distribution on the circle. An isotropic distribution of neighbours would correspond to a uniform distribution of their angles on the circle, so we used the Hodges-Ajne test for uniformity as described and implemented in the Matlab Circular Statistics Toolbox [47]. We quantify the non-uniformity of these distributions using *trigonometric moments* $M_{\;p}=\langle M_{\;p}^{c},M_{\;p}^{s}\rangle$ as described by Jammalamadaka & SenGupta [48]. In figure 5, we examine how particularly relevant trigonometric moments vary by distance from the focal individual by looking at subsets of neighbours in concentric annuli. For details, see the electronic supplementary material, appendix F.

## Data Availability

The data are available on the Dryad Digital Repository: http://dx.doi.org/doi:10.5061/dryad.n02v6wwzz [49]. The code is available in the associated repository on Zenodo: http://dx.doi.org/doi:10.5281/zenodo.5787296 [50]. Additional information is provided in the electronic supplementary material [51].
